# Hydrodeoxygenation of 2,5-dimethyltetrahydrofuran over bifunctional Pt–Cs_2.5_H_0.5_PW_12_O_40_ catalyst in the gas phase: enhancing effect of gold†

**DOI:** 10.1039/d1ra09105k

**Published:** 2022-01-14

**Authors:** Hanan Althikrallah, Elena F. Kozhevnikova, Ivan V. Kozhevnikov

**Affiliations:** University of Liverpool, Department of Chemistry Liverpool L69 7ZD UK kozhev@liverpool.ac.uk; Department of Chemistry, King Faisal University, College of Science P.O. Box 400, Al-Ahsa 31982 Saudi Arabia halhekrallh@kfu.edu.sa

## Abstract

2,5-Dimethyltetrahydrofuran (DMTHF) is deoxygenated to *n*-hexane with >99% selectivity at mild conditions (90 °C, 1 bar H_2_ pressure, fixed-bed reactor) in the presence of the bifunctional metal-acid catalyst Pt–CsPW comprising Pt and Cs_2.5_H_0.5_PW_12_O_40_ (CsPW), an acidic Cs salt of Keggin-type heteropoly acid H_3_PW_12_O_40_. Addition of gold to the Pt–CsPW catalyst increases the turnover rate at Pt sites more than twofold, whereas the Au alone without Pt is not active. The enhancement of catalyst activity is attributed to PtAu alloying, which is supported by STEM-EDX and XRD analysis.

## Abstract 2

Addition of gold to the Pt–CsPW catalyst has an enhancing effect on the HDO of DMTHF, with a twofold increase of turnover rate at Pt sites.

## Graphical abstract

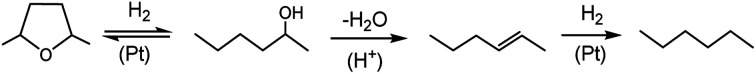

Biomass-derived furanic compounds are of interest as a renewable feedstock, which can be processed into a range of value-added chemicals and green fuels *via* catalytic hydroconversion.^1–8^ Hydrodeoxygenation (HDO) of furanic compounds using bifunctional metal–acid catalysis has been demonstrated to be an effective strategy to produce green fuels under mild conditions^3,4,6,8–12^ and references therein. The HDO over bifunctional metal-acid catalysts is much more efficient compared to the reaction over monofunctional metal catalysts.^11,12^ Previously, we have reported HDO of a wide range of oxygenates in the gas phase to produce alkanes in the presence of bifunctional catalysts comprising Pt, Ru, Ni and Cu as metal components and Keggin-type heteropoly acids, with their activity decreasing in the order Pt > Ru > Ni > Cu.^13,14^ Pt–CsPW comprising Pt and strongly acidic heteropoly salt Cs_2.5_H_0.5_PW_12_O_40_ (CsPW) has been reported to be a highly efficient catalyst for the HDO of 2,5-dimethylfuran (DMF) and 2,5-dimethyltetrahydrofuran (DMTHF) to produce *n*-hexane with 100% yield at 90–120 °C and ambient pressure.^11,12^ The HDO of DMTHF over Pt–CsPW occurs through a sequence of hydrogenolysis, dehydration and hydrogenation steps catalysed by Pt and proton sites of the bifunctional catalyst (Scheme 1). These include the ring opening of DMTHF to form 2-hexanol on Pt sites followed by its dehydration on proton sites of CsPW to hexene, which is finally hydrogenated to *n*-hexane on Pt sites.^12^ It is the facile dehydration of the secondary alcohol intermediate that drives the HDO process forward.^11,12^ The rate-limiting step is either the ring hydrogenolysis or 2-hexanol dehydration depending on the ratio of accessible surface metal and acid sites Pt/H^+^.^12^ Other platinum group metals such as Pd, Ru and Rh, that have high selectivity to ring hydrogenation rather than ring hydrogenolysis,^2,7^ have low activities in HDO of DMF and DMTHF.^11^

**Scheme 1:**
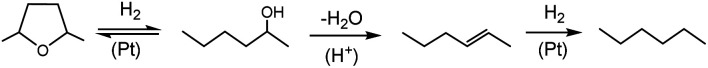
Reaction pathway for hydrodeoxygenation of DMTHF over Pt–CsPW.

Bimetallic PtAu and PdAu catalysts have been reported to have an enhanced performance in comparison to monometallic Pt and Pd catalysts,^15–31^ for example, in hydrogenation,^16,21,29^ hydrodesulphurisation,^27,28^ oxidation,^22,24^ isomerisation^15,19,30,31^ and other reactions.^17,18,20,25,26^ The enhancement of catalyst performance by addition of gold can be attributed to geometric (ensemble) and electronic (ligand) effects of the constituent elements in PtAu and PdAu bimetallic species.^25,26^

Here we looked at the effect of Au on the performance of Pt–CsPW catalysts in the HDO of DMTHF in the gas phase (see the ESI for experimental details†). The CsPW heteropoly salt is a well-known solid acid catalyst; it possesses strong proton sites, large surface area and high thermal stability (∼500 °C decomposition temperature).^9,32–34^ Supported bimetallic catalysts PtAu/SiO_2_ and PtAu/CsPW were prepared by co-impregnation of H_2_PtCl_6_ and HAuCl_3_ onto SiO_2_ and CsPW followed by reduction with H_2_ at 250 °C (ESI†). This method gives supported bimetallic PtAu nanoparticles of a random composition together with various Pt and Au nanoparticles.^15,16,31^ Information about the catalysts studied is given in Table 1.

**Table 1:** Catalyst characterisation

Catalyst	Surface area^a^ (m^2^ g^−1^)	Pore volume^b^ (cm^3^ g^−1^)	Pore diameter^c^ (Å)	*D* d	*d* e (nm)
Cs_2.5_H_0.5_PW_12_O_40_ (CsPW)	135	0.089	27		
6.5% Au/SiO_2_	257	1.01	157	0.019^f^	46^g^, 38^i^
4.7% Au/CsPW	103	0.048	33	0.016^f^	60^g^
6.4% Pt/SiO_2_	266	1.06	159	0.28 ± 0.04^h^	3.2^f^, 8.0^g^, 5^i^
6.0% Pt/CsPW	84	0.052	25	0.17 ± 0.03^h^	5.3^f^
6.6% Pt/5.9% Au/SiO_2_	240	1.08	179	0.29 ± 0.05^h^	3.1^f^
5.9% Pt/4.4% Au/CsPW	91	0.082	36	0.17 ± 0.04^h^	5.3^f^

a BET surface area.

b Single point total pore volume.

c Average BET pore diameter.

d Metal dispersion.

e Metal particle size.

f Calculated from the equation *d* (nm) = 0.9/*D*.

g Metal particle diameter from powder XRD (Scherrer equation).

h Pt dispersion determined by H_2_/O_2_ titration (average from three measurements); for PtAu catalysts, assumed negligible H_2_ adsorption on gold (see the ESI).

i From STEM.

Powder X-ray diffraction (XRD) has been widely used for the characterization of supported Au alloy catalysts.^26^ The XRD patterns for the silica-supported catalysts 6.4% Pt/SiO_2_, 6.5% Au/SiO_2_ and 6.6% Pt/5.9% Au/SiO_2_ are shown in Fig. 1. As expected, the 6.4% Pt/SiO_2_ and 6.5% Au/SiO_2_ catalysts display the fcc pattern of Pt and Au metal nanoparticles. The Pt peaks are broader than the Au peaks, which indicates a higher dispersion of Pt particles, with an average particle size of 8.0 nm for Pt and 46 nm for Au, which is in agreement with the STEM values (Table 1).

**Fig. 1:**
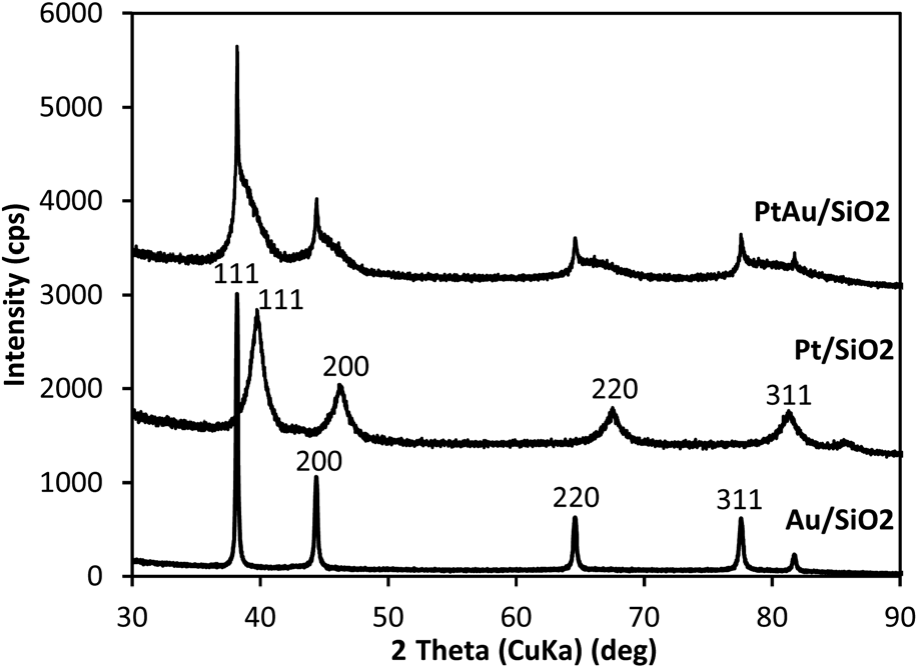
Powder XRD patterns of 6.4% Pt/SiO_2_, 6.5% Au/SiO_2_ and 6.6% Pt/5.9% Au/SiO_2_; the pattern for 6.6% Pt/5.9% Au/SiO_2_ shows broad [111], [200], [220] and [311] fcc PtAu alloy peaks in the range 38–40°, 44–48°, 65–68° and 78–81°, respectively.

The pattern for the 6.6% Pt/5.9% Au/SiO_2_ catalyst clearly shows the presence of PtAu bimetallic particles with broad [111], [200], [220] and [311] diffraction peaks of the fcc PtAu alloy between the corresponding diffractions of the pure metals in the range 38–40°, 44–48°, 65–68° and 78–81°, respectively.

Fig. 2 shows the high-angle annular dark field (HAADF) STEM images of the three silica-supported catalysts 6.4% Pt/SiO_2_, 6.5% Au/SiO_2_ and 6.6% Pt/5.9% Au/SiO_2_ with metal nanoparticles indicated as bright spots on the darker background. In the Pt/SiO_2_ catalyst, there are two populations: small Pt particles of 5 nm size and coalesced Pt particles of a larger size. The Au/SiO_2_ catalyst displays Au particles of spherical, rectangular and triangular morphology, with an average size of 38 nm. The bimetallic PtAu/SiO_2_ catalyst shows a high agglomeration and different kinds of morphology of metal particles.

**Fig. 2:**
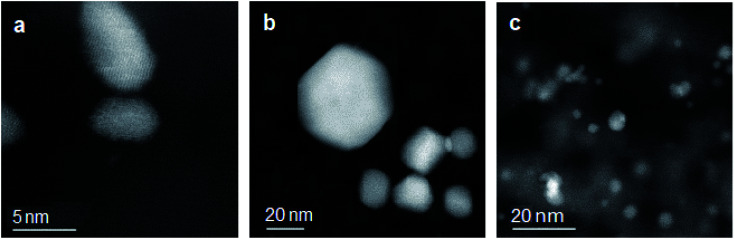
HAADF-STEM images of (a) 6.4% Pt/SiO_2_, (b) 6.5% Au/SiO_2_ and (c) 6.6% Pt/5.9% Au/SiO_2_ catalysts, showing noble metal nanoparticles as bright spots.

The energy dispersive X-ray spectroscopic analysis (EDX) of metal particles in the PtAu/SiO_2_ catalyst shows that these particles contain both platinum and gold. EDX elemental mapping clearly demonstrates that Pt and Au maps cover the same areas of PtAu/SiO_2_ catalyst (Fig. 3), indicating PtAu alloying with formation of a non-uniform bimetallic PtAu particles. More EDX mapping is presented in the ESI (Fig. S1†).

**Fig. 3:**
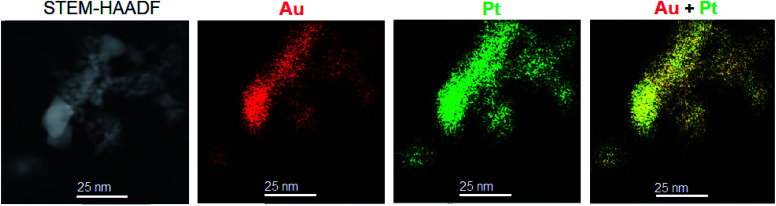
HAADF-STEM image of 6.6% Pt/5.9% Au/SiO_2_ catalyst and the corresponding STEM-EDX elemental maps showing the spatial distribution of Au (red) and Pt (green) in the sample.

STEM–EDX for CsPW-supported Pt, Au and PtAu catalysts has been reported elsewhere.^16^ These STEM images are difficult to analyse due to W, Pt and Au having similar large atomic numbers *Z* (74, 78, and 79, respectively). Crystalline CsPW containing 70 wt% of W displays a strong background which makes it difficult to discern smaller Pt and Au particles from the Z-contrast HAADF images and determine accurately metal particle size. Nevertheless, the STEM-EDX analysis indicates the presence of bimetallic PtAu particles in the PtAu/CsPW catalyst with a wide range of Pt/Au atomic ratios.^16^

Representative results for HDO of DMTHF in the presence of bifunctional metal-acid catalysts Pt–CsPW and PtAu–CsPW, which were used as physical mixtures of metal and acid components at similar Pt loadings, are shown in Table 2. The HDO reaction was carried out in flowing hydrogen at 90 °C and ambient pressure in a fixed-bed reactor (ESI†). The molar ratio of surface metal and proton sites in the catalysts was chosen low enough (Pt/H^+^ = 0.03–0.1) to ensure the reaction being limited by the DMTHF ring opening step.^12^ The density of surface Pt sites was estimated from the Pt dispersion (Table 1), the density of surface proton sites in CsPW was calculated assuming a cross section of the PW_12_O_40_^3−^ Keggin unit of 144 Å^32,33^ and the CsPW surface area of 135 cm^2^ g^−1^ (Table 1).

**Table 2:** Hydrodeoxygenation of DMTHF over bifunctional metal-acid catalysts^a^

Entry	Catalyst	Conversion (%)	TOF^b^ (h^−1^)	Product selectivity (% mol)	
				*n*-Hexane	2-Hexanol
1	CsPW	2.1			
2	4.7% Au/CsPW + CsPW	2.2			
3	6.0% Pt/CsPW + CsPW	8.6	70	98.6	0.7
4	5.9% Pt/4.4% Au/CsPW + CsPW	17	170	98.6	0.8
5	6.5% Au/SiO_2_ + CsPW	1.9			
6	6.4% Pt/SiO_2_ + CsPW	64	390	99.4	0.5
7	6.6% Pt/5.9% Au/SiO_2_ + CsPW	85	490	99.6	0.3
8	6.4% Pt/SiO_2_ + CsPW^c^	8.0	150	98.6	1.2
9	6.6% Pt/5.9% Au/SiO_2_ + CsPW^c^	13	260	98.5	0.7

a 0.20 g total catalyst weight (physical mixture of 0.020 g metal catalyst + 0.18 g CsPW), 0.6% Pt, 90 °C, 2.3 kPa DMTHF, 20 ml min^−1^ H_2_ flow rate, catalyst pre-treatment at 90 °C for 1 h in H_2_ flow, 1 h TOS.

b TOF values per Pt surface site, the contribution of Au and CsPW subtracted.

c Catalyst bed contained 0.005 g metal catalyst + 0.18 g CsPW; catalyst pre-treatment at 250 °C for 1 h in H_2_ flow.

In the absence of Pt, the CsPW alone (entry 1) and Au–CsPW (entries 2 and 5) showed a negligible activity (1.9–2.2% DMTHF conversion with practically no 2-hexanol and *n*-hexane formed). Physically mixed Pt–CsPW catalysts, Pt/CsPW + CsPW and Pt/SiO_2_ + CsPW (1 : 9 w/w), exhibited a high activity giving >99% *n*-hexane selectivity at 8.0 to 85% DMTHF conversion depending on the catalyst and reaction conditions, in agreement with the previous report.^12^ It should be noted that the catalyst based on Pt/SiO_2_ had almost 6-fold greater activity than the one based on Pt/CsPW in terms of turnover frequency (TOF) per surface Pt site (*cf*. entries 3 and 6), thus demonstrating a strong effect of Pt support.

As can be seen from Table 2, the addition of gold to Pt/CsPW and Pt/SiO_2_ caused a significant enhancement of catalyst activity, with DMTHF conversion increasing 1.3–2 times compared to the corresponding Pt only catalysts (*cf*. entries 3 and 4, 6 and 7, 8 and 9). As the gold alone was practically inactive, the increase in catalyst activity can be attributed to the enhancement of activity of the Pt sites. For the bimetallic PtAu catalysts, the TOF values at Pt sites increased 1.3–2.4 times as compared to the monometallic Pt catalysts (Table 2). The results at lower DMTHF conversions of 8–17% give a more accurate estimate of the TOF enhancement reaching 1.7–2.4 times (*cf*. entry 3 with 4 and 8 with 9). Previously, the gold enhancement on a similar scale has been reported for alkane isomerisation on PtAu–CsPW catalysts.^15,31^

The enhancement of catalyst activity by addition of gold has been attributed to geometric (ensemble) and electronic (ligand) effects of the constituent metals in PtAu bimetallic nanoparticles.^26^ The XRD and STEM-EDX data shown above clearly demonstrate PtAu alloying in the PtAu/SiO_2_ catalyst leading to the formation of bimetallic PtAu species. The same has also been reported for the PtAu/CsPW catalyst.^16^ Previously, it has been shown that the HDO of DMTHF on Pt–CsPW is a structure-sensitive reaction,^12^ hence the geometric effects may be expected to contribute to the gold enhancement. However, in order to prove the role of geometric and electronic effects as the cause of the gold enhancement, more accurate metal dispersion measurements complemented by spectroscopic characterisation will be required.

We also tested the performance of bifunctional PdAu/SiO_2_ + CsPW and PtPd/SiO_2_ + CsPW bimetallic catalysts under similar conditions in comparison to the corresponding monometallic Pd and Pt catalysts. However, no enhancement of activity was observed. This is in agreement with XRD analysis, which showed no distinct PdAu alloying in PdAu/SiO_2_ (Fig. S2 in the ESI†).

In conclusion, we have demonstrated that the addition of gold to the Pt–CsPW catalyst has an enhancing effect on the HDO of DMTHF, increasing the turnover rate at Pt sites more than twofold. The enhancing effect is attributed to PtAu alloying. The formation of bimetallic PtAu nanoparticles in the PtAu–CsPW catalyst is confirmed by STEM-EDX and XRD.

## Author contributions

The manuscript was written through contributions of all authors.

## Conflicts of interest

There are no conflicts to declare.

## Supplementary Material

RA-012-D1RA09105K-s001
