# Supplementary material for: Hydrodeoxygenation of 2,5-dimethyltetrahydrofuran over bifunctional Pt–Cs2.5H0.5PW12O40 catalyst in the gas phase: enhancing effect of gold
Source: RSC Adv. 2022 Jan 14;12(4):2287–91. doi: 10.1039/d1ra09105k (PMC8979146; doi:10.1039/d1ra09105k)
Supplement: RA-012-D1RA09105K-s001 [file RA-012-D1RA09105K-s001.pdf]

Electronic Supplementary Material (ESI) for RSC Advances.
This journal is © The Royal Society of Chemistry 2022

Hydrodeoxygenation of 2,5-dimethyltetrahydrofuran over bifunctional Pt–$Cs_{2.5}H_{0.5}PW_{12}O_{40}$ catalyst in the gas phase: Enhancing effect of gold

Hanan Althikrallah,[a,b] Elena F. Kozhevnikova,[a] Ivan V. Kozhevnikov[a*]

[a] *University of Liverpool, Department of Chemistry, Liverpool L69 7ZD, UK*

[b] *Department of Chemistry, College of Science, King Faisal University, P.O Box 400, Al-Ahsa, 31982, Saudi Arabia*

Electronic Supplementary Information

Chemicals and catalysts

Techniques

Catalyst testing

STEM–EDX images

XRD

Chemicals and catalysts. 2,5-Dimethyltetrahydrofuran (DMTHF, *cis/trans* isomer mixture, 96%), Cs_2CO_3 and $H_3PW_{12}O_{40}$ hydrate (99%) were purchased from Sigma-Aldrich. Aerosil 300 silica support ($S_{BET} \approx 300\ m^2g^{-1}$) was from Degussa.

CsPW was prepared according to the literature procedure[1] and vacuum dried at 150 $^oC/10^{-3}$ kPa for 1.5 h. Supported Pt and Au catalysts were prepared by wet impregnation of CsPW or Aerosil 300 silica (SiO_2) with aqueous solutions of H_2PtCl_6 or $HAuCl_4$, as described previously.[2–4] The preparation involved stirring the aqueous slurry at 50 ^oC for 2 h, rotary evaporation to dryness and reduction with H_2 flow at 250 ^oC for 2 h. Supported bimetallic PtAu catalysts were prepared similarly via co-impregnation of CsPW or SiO_2

* Corresponding author. Tel.: +44(0)1517942938
E-mail address: kozhev@liverpool.ac.uk (I. V. Kozhevnikov)

with H_2PtCl_6 and $HAuCl_4$ from aqueous solution with reduction by H_2 as above. Pt and Au loading in these catalysts was determined by ICP-OES. Physical mixtures of Pt, Au and PtAu catalysts with CsPW and SiO_2 were prepared by grinding the catalysts with CsPW and Aerosil 300 silica to 40–180 μm particle size. Information about the catalysts prepared is presented in Table 1 in the main text.

Techniques. The surface area and porosity of catalysts were determined from nitrogen physisorption measured on a Micromeritics ASAP 2010 instrument at −196 °C. Before measurement, the samples were evacuated at 250 °C. Inductively coupled plasma optical emission spectroscopy (ICP–OES) elemental analysis was carried out on a Spectro Ciros optical emission spectrometer. Powder X-ray diffraction (XRD) patterns of catalysts were recorded on a PANalytical Xpert diffractometer with CuKα radiation (λ = 1.542 Å) and attributed using the JCPDS database. High-angle annular dark-field scanning transmission electron microscopy (HAADF-STEM) images were obtained on a spherical aberration-corrected JEOL 2100 F microscope at an accelerating voltage of 200 kV. Energy dispersive X-ray spectroscopy (EDX) mapping was performed using JEOL Silicon Drift Detector DrySD100GV with a solid angle of up to 0.98 steradians from a detection area of 100 mm^2. The STEM–EDX work was performed in the Albert Crewe Centre for Electron Microscopy, a University of Liverpool Shared Research Facility (SRF).

Platinum dispersion, D, in supported Pt and PtAu catalysts defined as the Pt fraction at the surface of platinum particles, $D = Pt_s/Pt_{total}$, was measured on a Micromeritics TPD/TPR 2900 instrument using H_2/O_2 titration pulse method in a flow system at room temperature as described previously.[2–4] The D values were obtained assuming the stoichiometry of H_2 adsorption $Pt_sO + 1.5\ H_2 \rightarrow Pt_sH + H_2O$.[5,6] Adsorption of H_2 on PtAu catalysts was attributed entirely to platinum as Au did not adsorb H_2 under such

conditions.[2–4] The average diameter of Pt particles, d, was obtained from the empirical equation d (nm) = $0.9/D$.[6] The size of Pt and Au particles was also estimated from STEM images and XRD using the Scherrer equation.

Catalyst testing. The HDO of DMTHF was carried out in a gas-solid system at 90 ^{o}C in flowing H_2 at ambient pressure using a down-flow fixed-bed microreactor (9 mm internal diameter) fitted with an on-line GC analysis as described elsewhere.[7] DMTHF was fed by passing H_2 flow controlled by a Brooks mass flow controller through a saturator, which held the liquid substrate at 0 ^{o}C to maintain the chosen DMTHF partial pressure of 2,3 kPa. The gas feed entered the reactor at a flow rate of 20 ml min^{-1}. The catalyst bed (0.20 g total weight) contained a uniform physical mixture of powdered catalyst components (45-180 μm particle size). Prior to reaction, the catalysts were pre-treated in situ for 1 h at the reaction temperature in H_2 flow unless stated otherwise. Reactions were carried for 3 h time on stream (TOS); each run was reproduced at least twice. Product selectivity was defined on the carbon basis as moles of product formed per one mole of substrate converted and quoted in mole per cent. The mean absolute percentage error in conversion and selectivity was ≤ 5% and the carbon balance was maintained within 95%. The initial reaction rate (R) based on total Pt weight in the catalyst was calculated as $R = XF/W$ (mol h^{-1}g^{-1}), where X is the fractional conversion at 1 h TOS, F (mol h^{-1}) is the inlet molar flow rate of DMTHF and W is the weight of Pt (g) in the catalyst. The initial turnover frequency (TOF) was calculated as the initial reaction rate per surface Pt site, TOF = R/D (h^{-1}), using the values of Pt dispersion D from Table 1.

STEM–EDX images

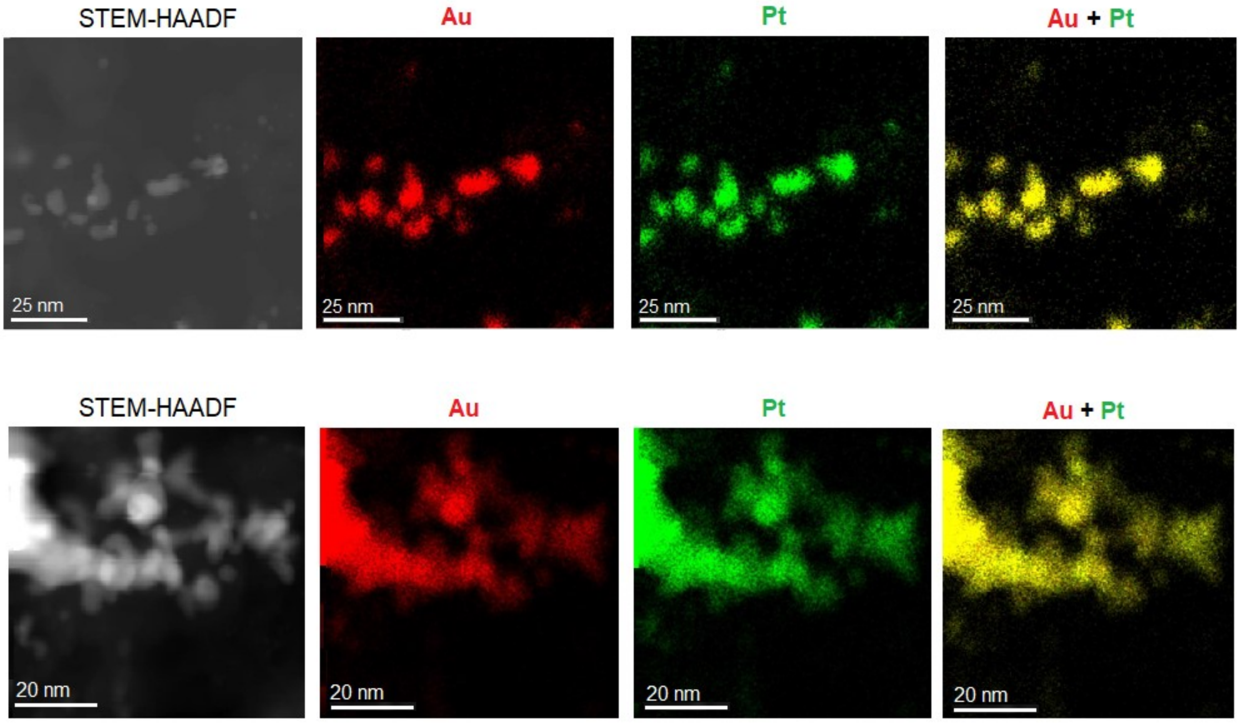

Fig. S1 HAADF–STEM images of 6.6%Pt/5.9%Au/SiO_2 catalyst and the corresponding STEM–EDX elemental maps showing the spatial distribution of Au (red) and Pt (green) in the sample.

XRD

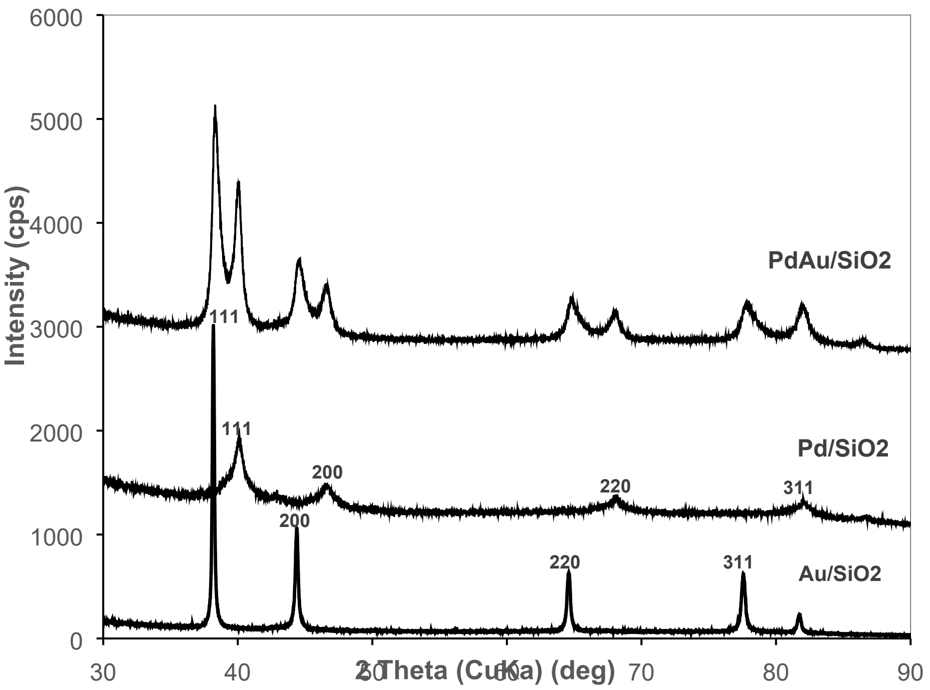

Fig. S2 Powder XRD patterns: 6.5%Au/SiO_2, 6.0%Pd/SiO_2 and 5.6%Pd/5.9%Au/SiO_2.

References

1 Y. Izumi, M. Ono, M. Kitagawa, M. Yoshida and K. Urabe, Silica-included heteropoly compounds as solid acid catalysts, *Microporous Mater.* 1995, **5,** 255–262.

2 O. Poole, K. Alharbi, D. Belic, E. F. Kozhevnikova and I. V. Kozhevnikov, Hydrodeoxygenation of 3-pentanone over bifunctional Pt-heteropoly acid catalyst in the gas phase: Enhancing effect of gold. *Appl. Catal. B* 2017, **202,** 446–453.

3 A. Alazman, D. Belic, E. F. Kozhevnikova and I. V. Kozhevnikov, Isomerization of n-hexane over bifunctional Pt-heteropoly acid catalyst: Enhancing effect of gold, *J. Catal.* 2018, **357**, 80-89.

4 A. Alazman, D. Belic, A. Alotaibi, E. F. Kozhevnikova and I. V. Kozhevnikov, Isomerization of cyclohexane over bifunctional Pt-, Au-, and PtAu-heteropoly acid catalysts, *ACS Catal.* 2019, **9**, 5063−5073.

5 J. E. Benson and M. Boudart, Hydrogen-oxygen titration method for the measurement of supported platinum surface areas, *J. Catal.* 1965, **4,** 704–710.

6 J. E. Benson, H. S. Hwang and M. Boudart, Hydrogen-oxygen titration method for the measurement of supported palladium surface areas, *J. Catal.* **1973,** *30*, 146-153.

7 H. Althikrallah, E. F. Kozhevnikova and I. V. Kozhevnikov, Hydrodeoxygenation of 2,5-dimethyltetrahydrofuran over bifunctional metal-acid catalyst Pt–$Cs_{2.5}H_{0.5}PW_{12}O_{40}$ in the gas phase: Kinetics and mechanism, *Mol. Catal.* 2021, **510,** 111711.
